# Non-small Cell Lung Cancer with Multiple Brain Metastases Treated with Radiosurgery and Erlotinib: A Case Report

**DOI:** 10.7759/cureus.2003

**Published:** 2017-12-29

**Authors:** Bilgehan Sahin, Teuta Mustafayev, Gokhan Aydin, Gorkem Gungor, Bulend Yapici, Banu Atalar, Enis Ozyar

**Affiliations:** 1 Acibadem University Acibabem Maslak Hospital; 2 Radiation Oncology, Acibadem University Acibabem Maslak Hospital, Turkey; 3 Acibadem University Acibabem Maslak Hospital, Turkey

**Keywords:** multiple brain metastases, cyberknife radiosurgery, erlotinib

## Abstract

Brain metastases are commonly seen complications in non-small cell lung cancer (NSCLC) patients. The incidence of brain metastases is increasing as a result of more effective systemic targeted therapies with prolonged survival. The prognosis is usually poor, and up to six months of median survivals were reported with different therapeutic options. Here, we present an NSCLC case with multiple brain metastases treated with radiosurgery and systemic erlotinib therapy with prolonged survival. The use of tyrosine kinase inhibitors (TKI) in conjunction with either stereotactic radiosurgery or whole brain radiotherapy is not well established in terms of efficiency and toxicity. This reported case had an excellent response with a tolerable toxicity profile from the combination of either therapies.

## Introduction

The incidence of brain metastases (BM) in non-small cell lung cancer (NSCLC) patients has elevated up to 20-40% of cases [1, 2]. Improved imaging modalities and enhanced systemic therapeutic options for the treatment of extracranial disease has led to prolonged survival with higher incidence of BM. In historical series, whole brain radiotherapy (WBRT) was used as the mainstay of the treatment and utilized in a palliative manner combined with corticosteroids and anticonvulsants in a majority of cases; generally, radiosurgery was reserved for selected cases [3]. As radiosurgery techniques improve and more targeted therapies such as tyrosine kinase inhibitors (TKIs) are generated, more therapeutic options are available. Surgery, stereotactic radiosurgery (SRS), WBRT, chemotherapy, and TKIs can be used solely or in combination [4].

## Case presentation

**Figure 1:**
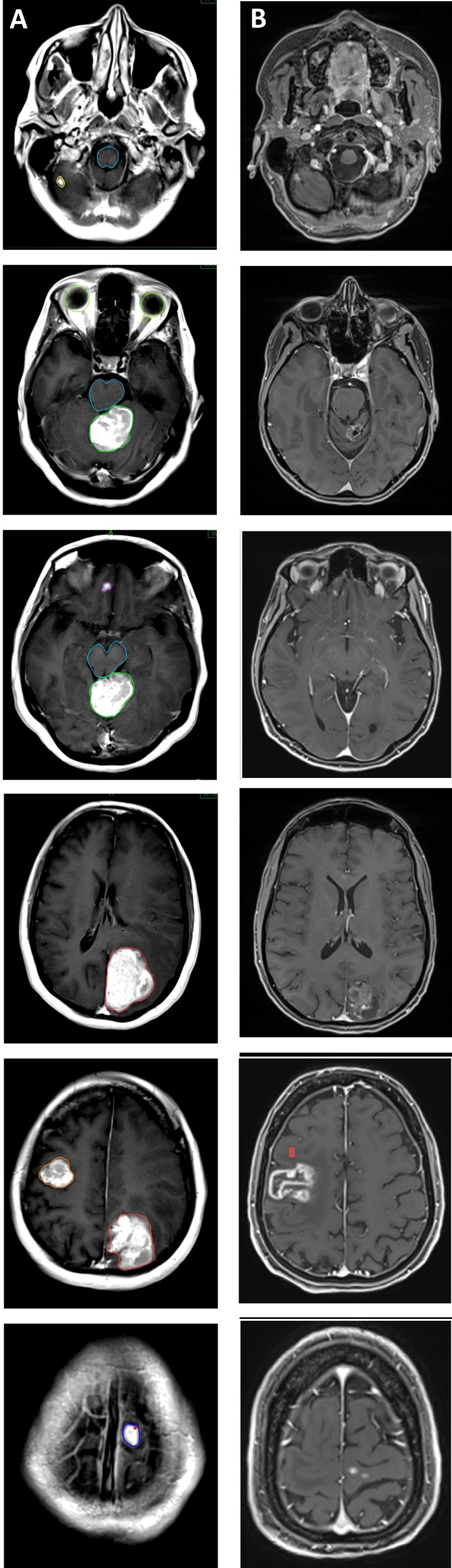
Magnetic resonance imaging scans before and after stereotactic radiosurgery A: Initial cranial contrast-enhanced T1 axial magnetic resonance scan (blue: brainstem; other colours denote different metastases). B: August 2017 dated contrast-enhanced T1 axial magnetic resonance scan, two years and seven months after stereotactic radiosurgery, illustrating regression in the five lesions and progression in the right frontal lesion, denoted by the red arrow.

We present a 47-year-old woman who had balance problems for three months. In January 2015, imaging techniques  revealed multiple brain metastases and a right lung malignant lesion with mediastinal and supraclavicular lymph nodes. A supraclavicular biopsy revealed an adenocarcinoma histopathology with thyroid-specific transcription factor-1 (TTF-1) and cytokeratin-7 (CK-7) positivity. She had imbalance with gait disorder and no other complaints. She was admitted to our hospital for the treatment of the brain metastases. A cranial magnetic resonance imaging (MRI) revealed that she had six metastases. Two of them were large in diameter and one of them was creating pressure on the brainstem with an edematous zone surrounding the core lesion (Figure 1A). For this reason, she was advised to have WBRT first and robotic radiosurgery boost one month later according to the response. The patient did not agree to undergo WBRT because of concerns and anxiety about potential side effects. Between January 22, 2015 and January 28, 2015, the patient had robotic radiosurgery for her six brain lesions. Two lesions were treated with 25 Gy in five fractions and the remainder were treated with 18 Gy in one fraction. Her imbalance and gait disorder improved rapidly. As the epidermal growth factor receptor (EGFR) was positive (subtype of exon 19 or 21 deletion was not known), the patient started to use the first line TKI; erlotinib (Tarceva©, Roche Genentech Inc., CA, USA) 150 mg per day orally as a systemic therapy.

The patient continued treatment with erlotinib without any complaints for two years and four months. In May 2017, 29 months after radiosurgery, the patient developed sudden left upper extremity paresis. A multiparametric cranial MRI including perfusion, diffusion MRI, and MR spectroscopy demonstrated that all treated lesions had regressed, but a lesion at the right frontal lobe, 24 x 33 mm in diameter, had increased vascularization peripherally and had progressed, and it was accepted as a recurrence of a previously irradiated lesion (Figure 1B). Erlotinib was discontinued and 8 mgr/day of dexamethasone was started. The left upper extremity weakness got better, but it did not fully recover. A positron emission tomography - computed tomography (PET-CT) revealed a lesion at the right upper lobe and upper mediastinal lymph nodes with increased fluorodeoxyglucose (FDG) uptake. Surgery and radiosurgery options were described to the patient. Between August 17, 2017 and August 23, 2017 the recurrent lesion was treated with a total dose of 25 Gy in five fractions with robotic radiosurgery. Medical oncology consultation and histopathology revision for EGFR and programmed death-ligand 1 (PD-L1) were advised for further systemic therapy. After two years and 10 months from the first radiosurgery session, the patient is still alive with the disease.

## Discussion

This case represents the long term survival of a patient with multiple large metastatic lesions with the combined use of radiosurgery and a first line TKI, Erlotinib. As a classical approach, WBRT with anti-edematous treatment was the main choice of treatment [3]. As more efficient systemic treatments appear, survival complicated by neurotoxicity and cognitive problems become more important. The use of TKI and radiotherapy in combination is not well established. Sperduto, et al. compared WBRT and SRS alone, with the combination of temozolomide (TMZ) or erlotinib in a radiation therapy oncology group (RTOG) phase III trial. The median survival times were 13.4, 6.3, and 6.1 months for WBRT+SRS, WBRT+SRS+TMZ, and WBRT+SRS+erlotinib, respectively. The performance status deterioration rates at six months increased from 53% to 86% with the addition of erlotinib (p<.001). The rates of Grade >2 toxicity related to treatment were increased from 11% to 49% with the addition of erlotinib. Despite the fact that the study was closed early due to low accrual of patients, the data suggests increased toxicity with the deleterious effect on the survival in unknown EGFR status [5]. In contrast to the previous study, Bai, et al. studied EGFR mutation-positive NSCLC patients with BM retrospectively. The disease control rate (DCR) was 87.2% and the median overall survival (OS) was 13.6 months. Only 11.5% of the patients observed Grade 3-4 toxicities including rashes, hepatotoxicity, and diarrhea. No patient discontinued TKI treatment due to increased toxicity [6].

The most efficient sequence of the treatments is also not well-defined. Magnuson, et al. studied upfront WBRT, SRS, and TKI in 351 EGFR-mutant patients. The median OS was 46, 30, and 25 months for the upfront SRS, WBRT, and EGFR-TKI, respectively (p<.001). Upfront SRS had better OS and better DCR compared to upfront WBRT and EGFR-TKI [7]. Similarly, a meta-analysis of 12 studies reported that upfront radiotherapy, either WBRT or SRS, improved OS in EGFR-mutant NSCLC patients [8]. The possible reason for more enhanced response and tumor control assumed that the blood-brain barrier (BBB) very intensively limits EGFR-TKI concentration in BM. Earlier SRS or WBRT disrupts BBB locally and gives way to increased doses of EGFR-TKI in the metastatic brain tissue [9].

In multiple BM, deciding whether the best radiotherapy option is WBRT or SRS is another issue. This case was initially advised to have WBRT and after the patient's refusal of WBRT, SRS was used as the primary radiotherapy treatment. Historically in patients with multiple BM, WBRT was the initial choice and SRS was reserved for fewer metastases with more favorable features. However, a multi-institutional prospective study reported that OS for 5-10 metastatic patients was non-inferior for patients with two to four BM [10]. This case represents that upfront SRS provides a path to long survival with a less toxicity profile by avoiding upfront WBRT.

## Conclusions

SRS in combination with erlotinib appears to have better results compared to WBRT or EGFR-TKI alone in efficiency in tumor response and tolerability in the toxicity profile. In this case, we present an NSCLC patient with large multiple BM with excellent initial tumor response and a recurrent brain metastatic lesion over 29 months after treatment, with prolonged survival and tolerable side effects.
